# Accelerated Photogenerated Charge Separation Driven Synergistically by the Interfacial Electric Field and Work Function in Z‐Scheme Zn‐Ni_2_P/G‐C_3_N_4_ for Efficient Photocatalytic Hydrogen Evolution

**DOI:** 10.1002/EXP.20240189

**Published:** 2025-08-05

**Authors:** Qian Chen, Jianfeng Huang, Dewei Chu, Liyun Cao, Xiaoyi Li, Yong Zhao, Yijun Liu, Junle Dong, Liangliang Feng

**Affiliations:** ^1^ School of Materials Science and Engineering International S&T Cooperation Foundation of Shaanxi Province Xi'an Key Laboratory of Green Manufacture of Ceramic Materials Shaanxi University of Science and Technology Xi'an China; ^2^ School of Materials Science and Engineering The University of New South Wales Sydney New South Wales Australia; ^3^ Guangxi Monalisa New Material Co., Ltd., Wu Zhou Guangxi P. R. China; ^4^ Guangdong Mona Lisa Group Co., Ltd. Foshan Guangdong P. R. China

**Keywords:** Z‐scheme heterojunction, g‐C_3_N_4_, interfacial electric field, work function, hydrogen evolution

## Abstract

The design of green and low‐cost Z‐scheme heterojunctions with the interfacial electric field (IEF) is of prime importance to their photocatalytic hydrogenation performance and practical application. In this work, we construct a novel Z‐scheme heterojunction photocatalyst comprised of Zn‐Ni_2_P/g‐C_3_N_4_ nanosheets for hydrogen evolution reaction (HER). Experimental results and density functional theory calculations demonstrate that the construction of Z‐scheme Zn‐Ni_2_P/g‐C_3_N_4_ heterostructure not only promotes the generation of IEF directing from Zn‐Ni_2_P to g‐C_3_N_4_, along with work function, accelerating the photogenerated charge separation in Zn‐Ni_2_P/g‐C_3_N_4_, but also leads to the upshift of the p‐band state density in Zn‐Ni_2_P/g‐C_3_N_4_, favorable for the H* adsorption toward HER. The Zn‐Ni_2_P/g‐C_3_N_4_ photocatalyst demonstrated excellent photocatalytic HER activity, with a hydrogen production rate of up to 1077 µmol g^−1^ h^−1^ and a stability of 49 h. Our findings provide a new method to enhance the separation of photogenerated charges. This improvement boosts the photocatalytic properties of solar‐driven materials and devices.

## Graphical abstract

The design of green and low‐cost Z‐scheme heterojunctions with the interfacial electric field is of prime importance to their photocatalytic hydrogenation performance and practical application. This work synthesizes Zn‐Ni_2_P/g‐C_3_N_4_ Z‐scheme heterojunction with a strong interfacial electric field for upgraded photocatalytic hydrogen evolution.

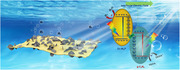

## 1 Introduction

The energy crisis will threaten our existence due to our over‐exploitation of non‐renewable fossil energy and the ever‐growing population. It is now undeniable that joint action is needed to develop long‐term solutions to the looming energy crisis [1, 2, 3]. Hydrogen energy is widely considered an ideal alternative to traditional fossil fuels because of its zero carbon emissions and high energy density. The exploitation of low‐carbon and environmentally friendly methods to prepare hydrogen gas on a large scale is a prerequisite for the industrial use of hydrogen energy [4, 5, 6]. The solar‐driven or electric‐driven hydrogen evolution reaction (HER) by water‐splitting technology provides a clean and renewable path for green hydrogen production.

Inspired by natural photosynthesis, semiconductor‐driven photocatalysis has been widely explored for the sustainable generation of clean molecular hydrogen. Photocatalytic water splitting can be deemed as a “Holy Grail” way to develop renewable hydrogen in the future energy structure because it converts natural solar energy into chemical energy in the form of H_2_. The rational design of an effective photocatalyst is highly pivotal because it directly speeds up the reaction kinetics of H_2_ evolution during the photocatalytic process [7, 8, 9, 10, 11]. As is well known that g‐C_3_N_4_ is currently one of the most popular 2D materials for photocatalytic water splitting on account of its suitable band structure, including a narrow band gap of about 2.7 eV and suitable band positions. Nevertheless, the efficiency of water splitting with g‐C_3_N_4_ is seriously restricted by the limited light‐response capability, insufficient active sites, and rapid recombination rate of photogenerated charges [12, 13, 14, 15]. To address these concerns, several strategies have been developed to improve the photocatalytic activity of g‐C_3_N_4_‐based materials. These include morphological adjustment, doping modification, cocatalyst loading (such as precious metals), and the construction of heterojunctions [16, 17, 18]. Thereinto, constructing heterostructure is proven to be conductive to creating an effective photocatalytic system with favorable visible‐light‐response, efficient photogenerated charge separation, and great redox capability [19, 20].

Among traditional heterojunctions, type‐II heterojunctions can effectively prevent the recombination of photogenerated electron–hole pairs. This is achieved by spatially separating electrons and holes at the interface of the composite photocatalysts. However, this comes at the cost of reducing the redox capacity of both semiconductor catalysts [18, 19, 20, 21]. The Z‐scheme heterojunction photocatalyst is constructed of two semiconductor catalysts in a staggered mode, which can realize the separation of electron–hole pairs with powerful redox capacity. However, most Z‐scheme heterojunction photocatalysts display unfulfilling photocatalytic HER activity as a result of the slow separation rate of photogenerated charges [22, 23, 24, 25]. Some new systems of Z‐scheme heterojunctions have been developed to solve the above problems. It has been reported that when a heterostructure is formed between two semiconductors with different charge densities, the negative charge center and the positive charge center are induced to have a tight barrier‐free interface, thus generating an interfacial electric field (IEF) at the heterojunction to promote the spatially directed migration of photogenerated charge [26, 27, 28]. Under light irradiation, the efficient separation of photogenerated charges can be achieved by the synergy of heterojunction and IEF [29, 30, 31, 32]. Therefore, integrating g‐C_3_N_4_ with a well‐matched semiconductor with a suitable energy band and charge densities is of great significance for upgraded photocatalytic HER performance. Transition metal phosphides (TMPs) are a critical transition metal compound, which is usually formed by the P atoms entering the lattice of transition metal and then leading to its enriched electron property. Thus, TMPs possess good electrical conductivity and unique metallic characteristics [33, 34, 35, 36, 37, 38, 39, 40, 41, 42, 43, 44]. Recently, nickel phosphide has gained significant attention due to its remarkable electronic properties. Ni_2_P, a promising nickel‐based phosphide material, features a narrow band gap and high work function. Its tunable band gap provides sufficient redox potential to overcome the thermodynamic barrier. As a result, Ni_2_P has been widely used in water splitting catalysis [36, 37, 38, 39].

In this study, a novel Z‐scheme heterojunction of Zn‐doped Ni_2_P nanoparticles modified with g‐C_3_N_4_ nanosheets (denoted as Zn‐Ni_2_P/g‐C_3_N_4_) is successfully synthesized. It exhibits abundant active sites, enhanced light response intensity, and strong electronic coupling at the interface. Experimental results and density functional theory (DFT) calculations demonstrate that the construction of Z‐scheme heterostructured Zn‐Ni_2_P/g‐C_3_N_4_ not only promotes the generation of IEF directing from Zn‐Ni_2_P to g‐C_3_N_4_, along with work function, accelerating the photogenerated charge separation in Zn‐Ni_2_P/g‐C_3_N_4_, but also leads to the upshift of the p‐band state density in Zn‐Ni_2_P/g‐C_3_N_4_, favorable for the H* adsorption toward photocatalytic HER. As envisioned, the optimized Zn‐Ni_2_P/g‐C_3_N_4_ photocatalyst has an excellent hydrogenation performance of 1077 µmol g^−1^ h^−1^ under visible light, even more than Pt/g‐C_3_N_4_. The resulting Zn‐Ni_2_P/g‐C_3_N_4_ Z‐scheme heterojunction is constructed as an efficient photocatalytic HER catalyst mainly due to the IEF and strong coupling of electrons between Zn‐Ni_2_P and g‐C_3_N_4_, which greatly accelerates the separation of photogenerated carriers. This research offers a novel insight for enabling the separation of photogenerated charge to increase the photocatalytic capabilities of solar‐driven materials and devices.

## 2 Experimental Section

### 2.1 Materials

#### 2.1.1 Chemicals and Reagents

The used chemicals and reagents are given in the Supporting Information.

### 2.2 Experiment

**FIGURE 1:**
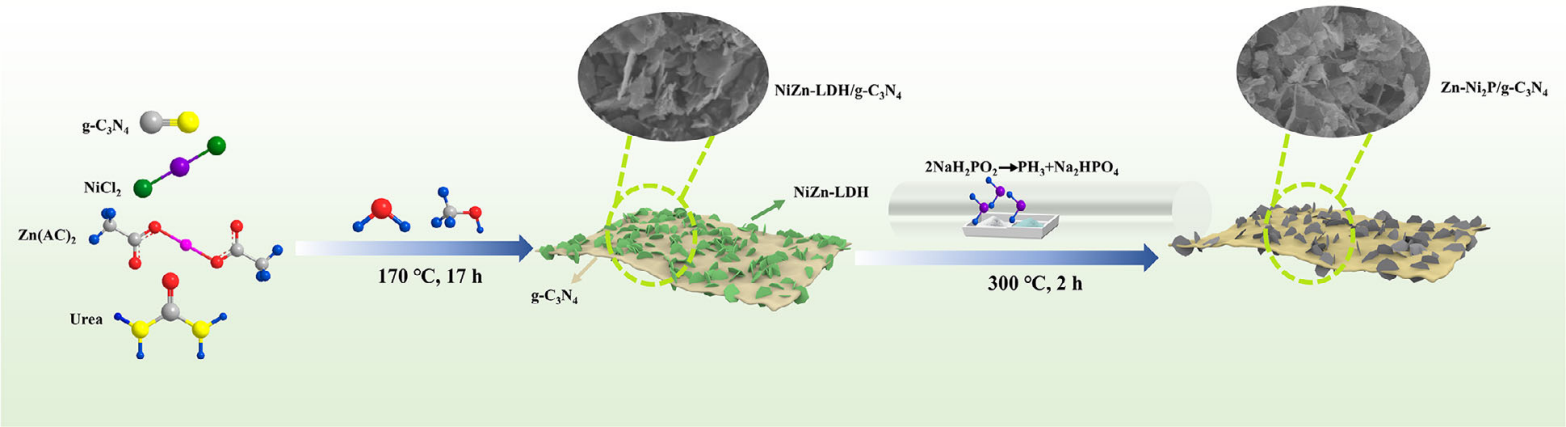
Schematic illustration for the construction of Zn‐Ni_2_P/g‐C_3_N_4_ heterojunction.

The Zn‐Ni_2_P/g‐C_3_N_4_ photocatalyst was synthesized via a simple hydrothermal reaction coupled with an in situ phosphating route, as depicted in Figure 1.

#### 2.2.1 Synthesis of the NiZn‐LDH

A mixture of Zn(AC)_2_·2H_2_O (0.1 mmol), NiCl_2_·6H_2_O (0.45 mmol), and urea (3 mmol) was sequentially dissolved in 60 mL of a methanol‐water solution (with a water‐to‐methanol volume ratio of 2:3) and stirred at room temperature for 30 min. The homogeneous solution was then transferred into a 100 mL Teflon‐lined stainless‐steel autoclave and subjected to hydrothermal treatment at 170°C for 17 h in a circulating hot air oven. After natural cooling, the resulting precipitate was separated by centrifugation, washed multiple times with deionized water and ethanol, and dried at 60°C for 12 h. The final product, a pale green powder, was labeled as NiZn‐LDH.

#### 2.2.2 Synthesis of the NiZn/G‐C_3_N_4_

The preparation of NiZn/g‐C_3_N_4_ followed a procedure similar to that of NiZn‐LDH. Zn(AC)_2_·2H_2_O (0.1 mmol), NiCl_2_·6H_2_O (0.45 mmol), urea (3 mmol), and varying amounts of g‐C_3_N_4_ (25, 50, 75, and 100 mg) were sequentially dissolved in 60 mL of a methanol–water mixed solvent (with a water‐to‐methanol volume ratio of 2:3), and stirred at ambient temperature for 30 min. The resulting solution was transferred into a 100 mL Teflon‐lined stainless steel autoclave and maintained at 170°C in an oven for 17 h. After cooling naturally to room temperature, the precipitate was collected by centrifugation, washed repeatedly (over six times) with deionized water and ethanol, and dried at 60°C for 12 h. The obtained powders were labeled as NiZn/g‐C_3_N_4_(1:1), NiZn/g‐C_3_N_4_(1:2), NiZn/g‐C_3_N_4_(1:3), and NiZn/g‐C_3_N_4_(1:4), corresponding to the respective g‐C_3_N_4_ contents.

#### 2.2.3 Synthesis of the Zn‐Ni_2_P/G‐C_3_N_4_

Zn‐Ni_2_P/g‐C_3_N_4_ was synthesized using a method similar to that of Zn‐Ni_2_P. Initially, the phosphorus source and the NiZn/g‐C_3_N_4_ powder were placed separately in porcelain boats, positioned upstream and downstream inside a tube furnace. The mass ratio between the phosphorus source and the powder was maintained at 3:5. The tube furnace was heated to 300°C at a rate of 5°C per minute under a mixed gas atmosphere of 5% hydrogen in argon, and held at this temperature for 2 h. After cooling naturally to room temperature under the same protective gas atmosphere, the resulting sample was collected, ground, and washed thoroughly (more than six times) with deionized water and ethanol. Finally, the product was dried at 60°C for 12 h. The obtained black powder was labeled as Zn‐Ni_2_P/g‐C_3_N_4_.

## 3 Results and Discussion

### 3.1 Characterizations of Morphology and Structure

The micro‐morphology of the synthesized pure g‐C_3_N_4_, Zn‐Ni_2_P, and Zn‐Ni_2_P/g‐C_3_N_4_ (optimized samples) was analyzed using X‐ray diffraction (XRD), Fourier‐transform infrared spectroscopy (FTIR), scanning electron microscopy (SEM), transmission electron microscopy (TEM), and X‐ray photoelectron spectroscopy (XPS).

**FIGURE 2:**
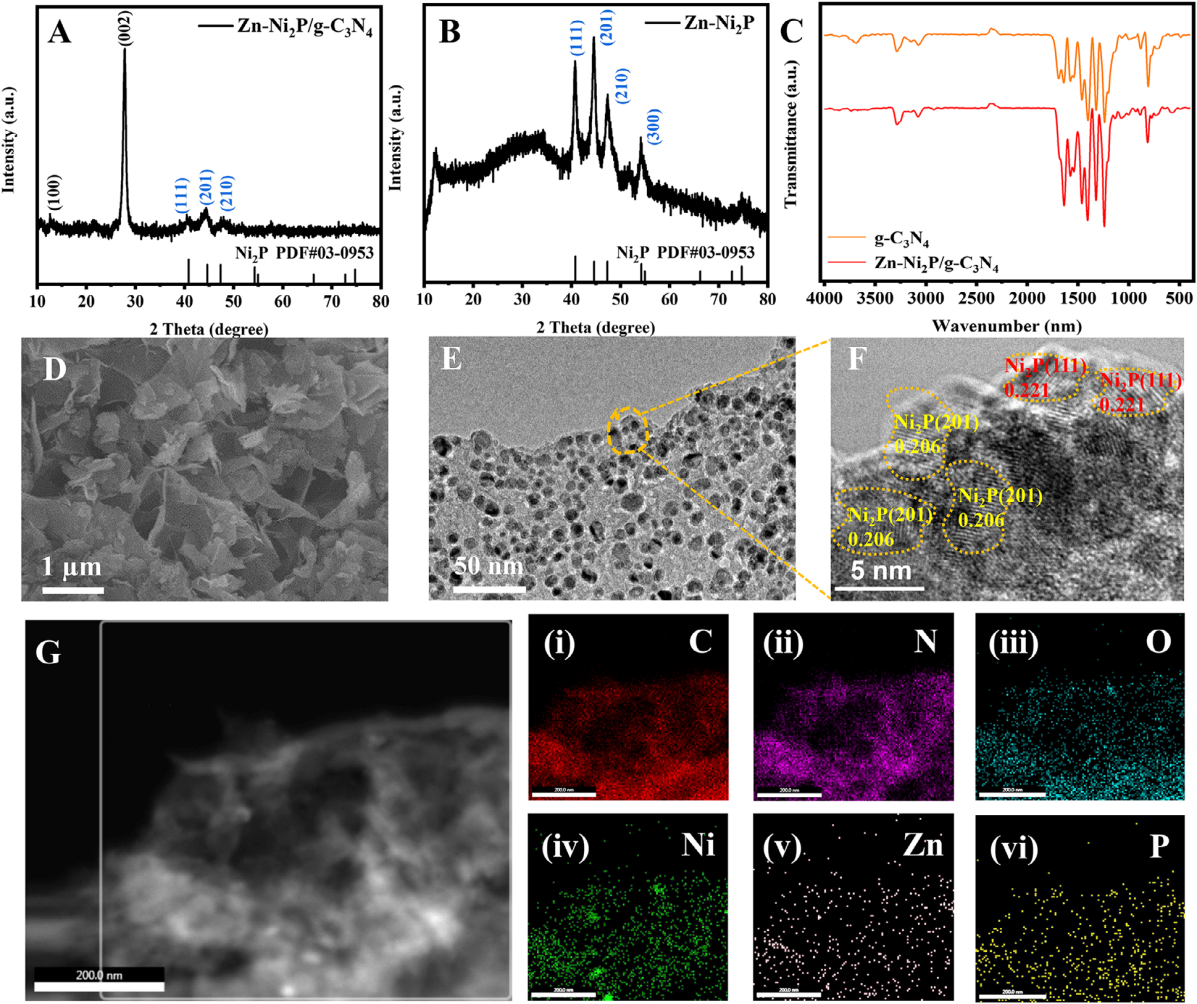
XRD patterns of (A) Zn‐Ni_2_P/g‐C_3_N_4_ and (B) Zn‐Ni_2_P. (C) FT‐IR spectra of Zn‐Ni_2_P/g‐C_3_N_4_ and Zn‐Ni_2_P. (D) SEM image of Zn‐Ni_2_P/g‐C_3_N_4_. (E) TEM image of Zn‐Ni_2_P/g‐C_3_N_4_. (F) High‐resolution TEM image of Zn‐Ni_2_P/g‐C_3_N_4_. (G) EDX elemental mapping images of Zn‐Ni_2_P/g‐C_3_N_4_.

To investigate the crystal structure, XRD measurements were performed, as shown in Figure 2A,B. In particular, Figure 2A presents the XRD pattern of Zn‐Ni_2_P/g‐C_3_N_4_, where a prominent peak at 27.7° corresponds to the characteristic diffraction of the conjugated aromatic system. The intensity and sharpness of this peak indicate a high degree of crystallinity in the synthesized g‐C_3_N_4_ material. In the Zn‐Ni_2_P/g‐C_3_N_4_ sample, in addition to the obvious peak of carbon nitride, the obvious diffraction peaks can be observed at 40.7° and 44.5° corresponding to (111) and (201) crystal planes of Ni_2_P (PDF# 03–0953) [45]. Figure 2B is the XRD pattern of Zn‐Ni_2_P. Its peak matches exactly with Ni_2_P (PDF# 03–0953), proving the successful preparation of the sample. Interestingly, relative to the Ni_2_P diffraction peaks (Figure S1), the (201) crystal plane in the Zn‐Ni_2_P diffraction peaks shows a preferential orientation. This indicates that Zn doping can induce the growth of the (201) crystal planes of Ni_2_P. To obtain more information about the sample structure, g‐C_3_N_4_ and Zn‐Ni_2_P/g‐C_3_N_4_ are tested with Fourier transform infrared spectrometry. The FTIR spectra of g‐C_3_N_4_ and Zn‐Ni_2_P/g‐C_3_N_4_ are shown in Figure 2C. Both samples showed typical FTIR spectra of g‐C_3_N_4_. At 807 cm^−1^ is the characteristic peak of the C─N heterocycle of the triazine ring. The peak observed in the area of 900–1800 cm^−1^ is the aromatic carbon nitrate heterocycle [46, 47, 48]. The wide peaks observed at 3100–3300 cm^−1^ may be primary and secondary amine groups and water molecules remaining on the g‐C_3_N_4_ surface [49]. Interestingly, no additional FTIR peaks were observed for Zn‐Ni_2_P/g‐C_3_N_4_, indicating that the P, Zn, and Ni complexes do not chemically bond with the g‐C_3_N_4_ heterocyclic framework. SEM images (Figure 2D) show that both g‐C_3_N_4_ and Zn‐Ni_2_P have nanosheet morphologies, which is further supported by the TEM results presented in Figure 2E–G. Comparing the TEM diagrams of Zn‐Ni_2_P and Ni_2_P (Figure S2C,F), we found that the particle size of Zn‐Ni_2_P is significantly smaller than Ni_2_P. This shows that Zn‐doping can play a limited‐domain role, inhibiting the growth of Ni_2_P nanoparticles and avoiding particle reunification. Figure 2F presents the HRTEM photo of Zn‐Ni_2_P/g‐C_3_N_4_. It shows the interplanar spacing of 0.206 and 0.221 nm that can be attributed to (201) and (111) crystal planes of Ni_2_P (PDF# 03–0953), respectively. Figure 2G shows the elemental mapping obtained by energy dispersive X‐ray spectroscopy (EDX), demonstrating the uniform distribution of C, N, O, Ni, Zn, and P elements.

**FIGURE 3:**
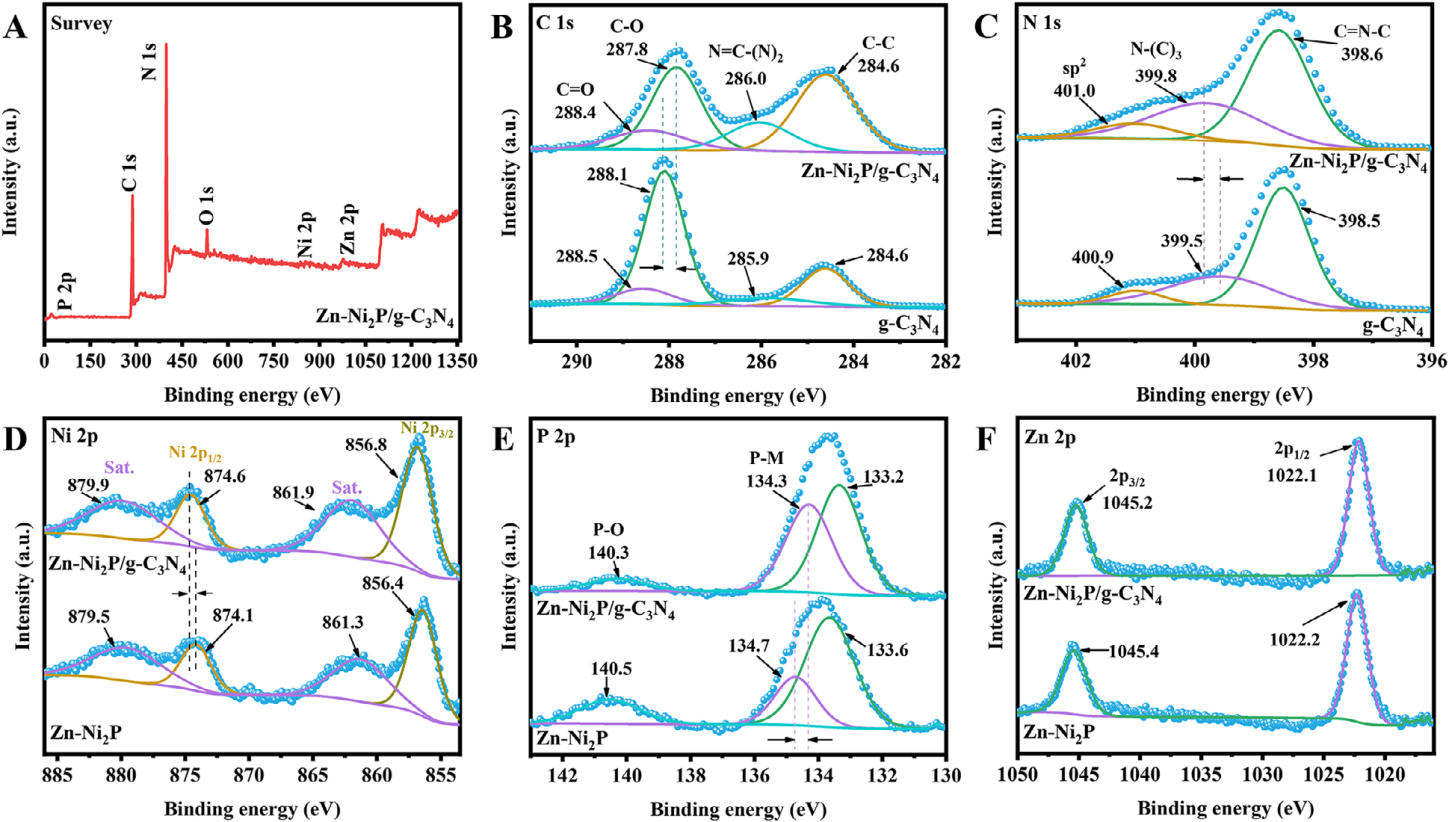
(A) XPS survey spectra, (B) C 1s spectra, (C) N 1s spectra, (D) Ni 2p spectra, (E) P 2p spectra, and (F) Zn 2p spectra of g‐C_3_N_4_, Zn‐Ni_2_P, and Zn‐Ni_2_P/g‐C_3_N_4_ composites.

The electronic structures and surface chemical states of g‐C_3_N_4_, Zn‐Ni_2_P, and Zn‐Ni_2_P/g‐C_3_N_4_ were further analyzed using XPS. As shown in Figure 3A, the XPS spectra of Zn‐Ni_2_P/g‐C_3_N_4_ reveal the presence of C, N, O, P, Zn, and Ni elements. For Zn‐Ni_2_P/g‐C_3_N_4_, the XPS spectra of C 1s display three peaks at 284.6, 286.0, 287.8, and 288.4 eV, corresponding to C─C, N═C─(N)_2_, C─O, and C═O of g‐C_3_N_4_, respectively (Figure 3B). The peaks of the N 1s spectrum of Zn‐Ni_2_P/g‐C_3_N_4_ (Figure 3C) at 398.6, 399.8, and 401.0 eV are assigned to C═N─C, N─(C)_3_, and sp^2^, respectively [50]. The XPS spectra of Ni 2p display peaks at 856.8, 861.9, 874.6, and 879.9 eV, corresponding to Ni 2p and sat. of Zn‐Ni_2_P, respectively (Figure 3D) [45]. The XPS spectra of P 2p display peaks in 133.2, 134.3, and 140.3 eV corresponding to P‐M and P‐O (Figure 3E) [50]. The XPS spectra of Zn 2p display peaks at 1022.1 and 1045.2 eV, corresponding to 2p_1/2_ and 2p_3/2_ in Zn‐Ni_2_P (Figure 3F). In the Zn‐Ni_2_P/g‐C_3_N_4_ composites, the C 1s and N 1s peaks shift to lower binding energies compared to pure g‐C_3_N_4_. Meanwhile, the Ni 2p, Zn 2p, and P 2p peaks shift to higher binding energies compared to pure Zn‐Ni_2_P. This shift indicates the formation of a well‐designed heterojunction with strong electronic coupling between Zn‐Ni_2_P and g‐C_3_N_4_. In addition, the surface charge is transferred from Zn‐Ni_2_p to the g‐C_3_N_4_ surface during the formation of the heterojunction, which increases the electron density of g‐C_3_N_4_.

### 3.2 Optical Performance and Electrochemical Analysis

**FIGURE 4:**
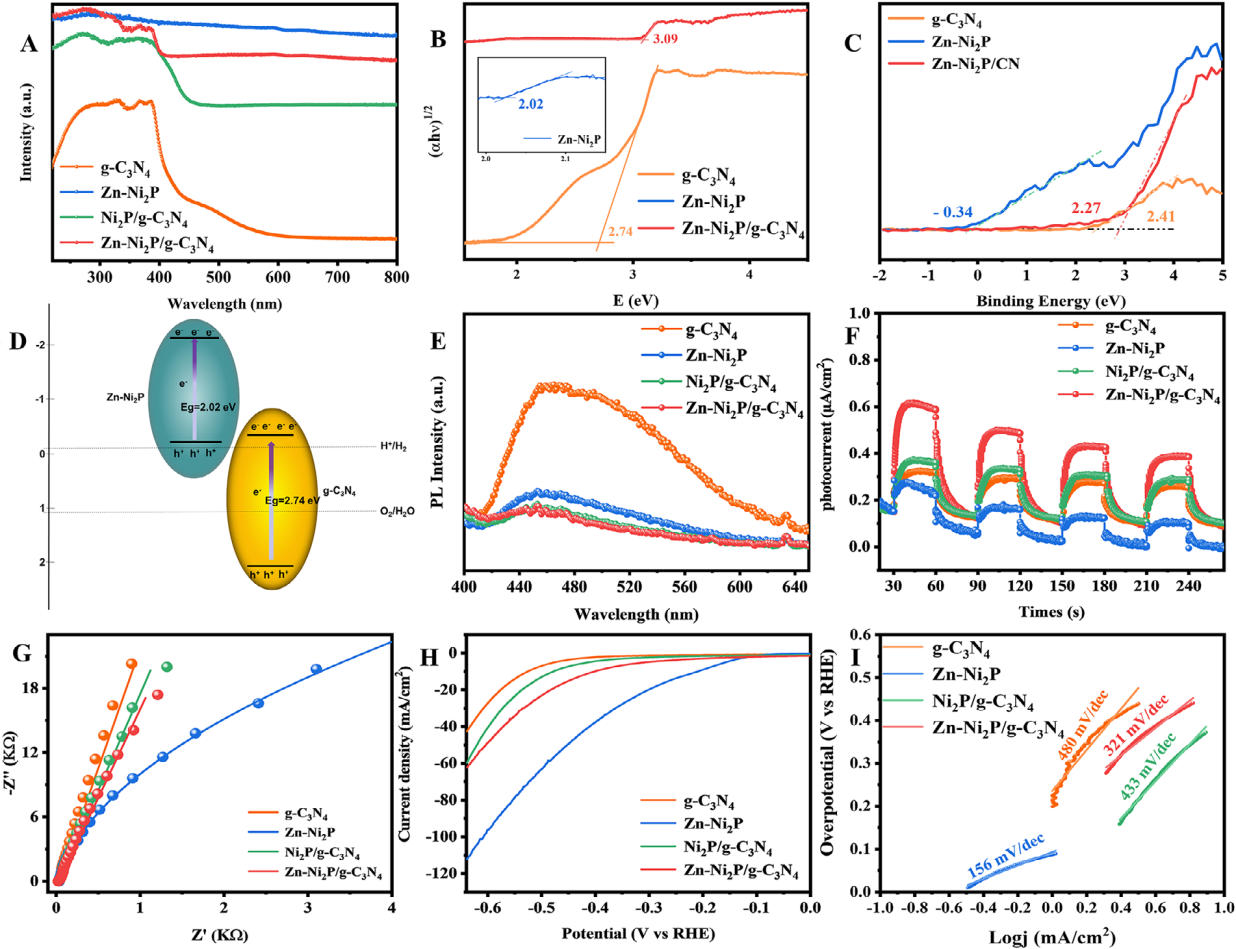
(A) UV–vis absorption spectra, (B) Kubelka–Munk function versus the energy of incident light plots of the as‐prepared samples and (C) valence band spectra obtained from g‐C_3_N_4_, Zn‐Ni_2_P and Zn‐Ni_2_P/g‐C_3_N_4_, (D) band structure of Zn‐Ni_2_P/g‐C_3_N_4_ sample, (E) steady‐state PL spectra, (F) transient photocurrent response, (G) EIS Nyquist plots under irradiation, (H) HER polarization curves and (I) Tafel slopes of g‐C_3_N_4_, Zn‐Ni_2_P, Ni_2_P/g‐C_3_N_4_, and Zn‐Ni_2_P/g‐C_3_N_4_.

The light response and absorption characteristics of photocatalysts are characterized by applying ultraviolet–visible diffuse reflectance spectroscopy (UV–vis DRS). As shown in Figure 4A, the Zn‐Ni_2_P/g‐C_3_N_4_ heterojunction sample shows an enhanced light absorption compared to g‐C_3_N_4_. The corresponding band gap energies of pure g‐C_3_N_4_, Zn‐Ni_2_P, and Zn‐Ni_2_P/g‐C_3_N_4_ samples are calculated by the transformed Kubelka–Munk function [51, 52]. As presented in Figure 4B, the pure g‐C_3_N_4_ has a band gap value of 2.74 eV, while Zn‐Ni_2_P and Zn‐Ni_2_P/g‐C_3_N_4_ have band gap energies of 2.02 and 3.09 eV. Therefore, we conducted the XPS valence band characterization (Figure 4C). The VB of g‐C_3_N_4_, Zn‐Ni_2_P, and Zn‐Ni_2_P/g‐C_3_N_4_ were measured to be 2.41, −0.34, and 2.27 eV, respectively. The schematic diagram of the energy bands of Zn‐Ni_2_P and g‐C_3_N_4_ is displayed in Figure 4D, suggesting the possible construction of a Z‐scheme heterojunction between Zn‐Ni_2_P and g‐C_3_N_4_ [53]. To study the separation and transfer efficiency of photogenerated carriers in Zn‐Ni_2_P/g‐C_3_N_4_, several tests were performed, including steady‐state photoluminescence spectroscopy (PL), photocurrent response (PC), and electrochemical impedance spectroscopy (EIS). Under excitation at 375 nm, pure g‐C_3_N_4_ exhibited the highest PL intensity, indicating a high rate of recombination of photogenerated carriers (Figure 4E). In contrast, Zn‐Ni_2_P/g‐C_3_N_4_ composites showed a significant reduction in PL intensity, confirming that combining g‐C_3_N_4_ with Zn‐Ni_2_P enhances charge separation. This result aligns with the improved photocatalytic performance [54, 55]. Figure 4E,G, corresponds to the PC and EIS plots of g‐C_3_N_4_, Ni_2_P/g‐C_3_N_4_, and Zn‐Ni_2_P/g‐C_3_N_4_, respectively. PC is generally used to characterize the photogenerated charge transport properties of photocatalysts. All photocatalysts exhibit a certain light flow response under intermittent cyclic illumination. The Zn‐Ni_2_P/g‐C_3_N_4_ sample also showed the highest photocurrent intensity among all samples (Figure 4F). Generally, a higher photocurrent indicates better separation efficiency of photogenerated charges. As shown in Figure 4G, the arc radius diameter of the EIS Nyquist diagram of Zn‐Ni_2_P/g‐C_3_N_4_ is much smaller than that of g‐C_3_N_4_, indicating that the combination of Zn‐Ni_2_P and g‐C_3_N_4_ is conducive to accelerating charge transfer and improving the interface charge transfer efficiency. In summary, these results demonstrate that forming a composite of Zn‐Ni_2_P with g‐C_3_N_4_ improves the separation and transport of photogenerated carriers, thereby enhancing the photocatalytic hydrogen HER. Linear scanning voltammetry (LSV) further confirms the role of Zn‐Ni_2_P in this process [56, 57, 58]. The HER activity of g‐C_3_N_4_, Ni_2_P/g‐C_3_N_4,_ and Zn‐Ni_2_P/g‐C_3_N_4_ was determined in alkaline medium (1.0 M KOH) by a three‐electrode system. Figure 4H reveals the LSV curves of various photocatalysts, and the Zn‐Ni_2_P/g‐C_3_N_4_ catalyst has a small overpotential, and the catalytic activity of the sample is affected by Zn‐doping. The Tafel slope is a critical parameter for evaluating the dynamics in HER. The Tafel slope plots of g‐C_3_N_4_ (480 mV·dec^−1^), Ni_2_P/g‐C_3_N_4_ (433 mV·dec^−1^), and Zn‐Ni_2_P/g‐C_3_N_4_ (321 mV·dec^−1^) were presented in Figure 4I. Overall, the introduction of Zn‐Ni_2_P effectively lowers the energy barrier for photocatalytic hydrogen evolution.

### 3.3 Photocatalytic H_2_ Evolution Reaction (HER)

**FIGURE 5:**
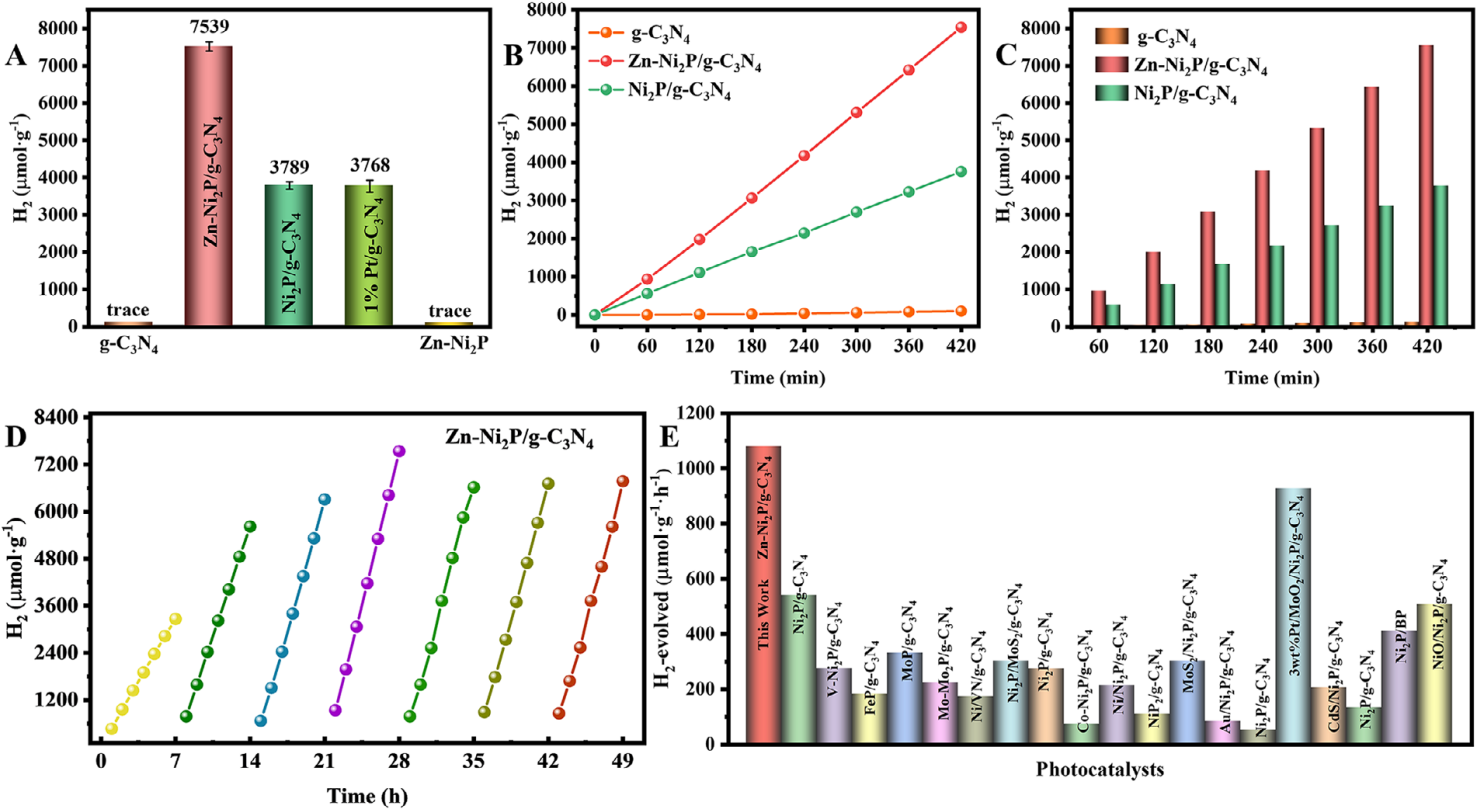
(A) The photocatalytic H_2_ evolution quantity in 7 h. (B,C) Time course of H_2_ production of various samples. (D) The H_2_ releasing yields of seven successive cycles. (E) Summary of the photocatalytic H_2_ evolution on g‐C_3_N_4_‐based photocatalysts under visible‐light.

The HER activity of the photocatalysts was tested under visible light using triethanolamine (TEOA) as a hole scavenger and without adding any other precious metals. As shown in Figure 5, Zn‐Ni_2_P alone showed no catalytic activity. The g‐C_3_N_4_ released only a trace amount of H_2_ due to the rapid recombination of photogenerated carriers. To further reveal the effect of Zn‐Ni_2_P on photocatalytic HER activity, several control tests are given in Figure S3A. The amount of H_2_ released from Zn‐Ni_2_P/g‐C_3_N_4_ changes with the added amount of Zn‐Ni_2_P. Zn‐Ni_2_P/g‐C_3_N_4_ obtains the highest hydrogen evolution activity when the synthesis mass ratio of Zn‐Ni_2_P to g‐C_3_N_4_ is 1:3. After anchoring Ni_2_P on the matrix of g‐C_3_N_4_, Ni_2_P has a metal‐like electron transfer rate and can be the HER site and significantly improving the photocatalytic HER efficiency of Ni_2_P/g‐C_3_N_4_ composites. The optimal hydrogen production rate of the Ni_2_P/g‐C_3_N_4_ composite is 3758 µmol·g^−1^. The results show that the close contact between g‐C_3_N_4_ and Ni_2_P accelerates charge transfer at the contact surface, allowing more photogenerated electrons can participate in the reaction. Zn doping significantly enhanced the photocatalytic hydrogen evolution performance, with the maximum hydrogen production reaching 7539 µmol g^−1^. The apparent quantum efficiency of Zn‐Ni_2_P/g‐C_3_N_4_ under 420 nm illumination was as high as 8.9%. Furthermore, as shown in Figure S3B, under AM 1.5 illumination, the hydrogen evolution rate of Zn‐Ni_2_P/g‐C_3_N_4_ reached 13,969 µmol g^−1^, about 1.85 times higher than that under visible light alone. Notably, this performance surpasses most reported g‐C_3_N_4_‐based photocatalysts (Figure 5E and Table S1).

The performance of HER is significantly improved due to the modulation of the catalyst structure by Zn doping. Similarly, the stability of photocatalysts is a key factor in assessing their performance. Figure 5D shows the results of a long‐term photocatalytic hydrogen evolution stability test. Zn‐Ni_2_P/g‐C_3_N_4_ was stable over at least 49 h of cycling, with hydrogen evolution activity staying nearly unchanged. XRD patterns before and after the cycling test (Figure S4) showed no significant changes in the characteristic peaks. Additionally, SEM images confirmed that the nanoparticle structure of Zn‐Ni_2_P/g‐C_3_N_4_ has been well maintained after cycling. At the same time, according to HRTEM images, Zn‐Ni_2_P nanoparticles can still be found. In conclusion, after photocatalysis of HER, the crystal structure and microstructure of Zn‐Ni_2_P/g‐C_3_N_4_ are almost unchanged. As shown in Figure S4E–I, the peak position and intensity of C, N, Ni, Zn, and P elements remained unchanged compared to the initial Zn‐Ni_2_P/g‐C_3_N_4_ photocatalyst. The results demonstrate that the composite photocatalyst has high stability, which is attributed to the formation of a strong heterojunction between g‐C_3_N_4_ and Zn‐Ni_2_P.

### 3.4 DFT Calculations and Photocatalytic Mechanism

**FIGURE 6:**
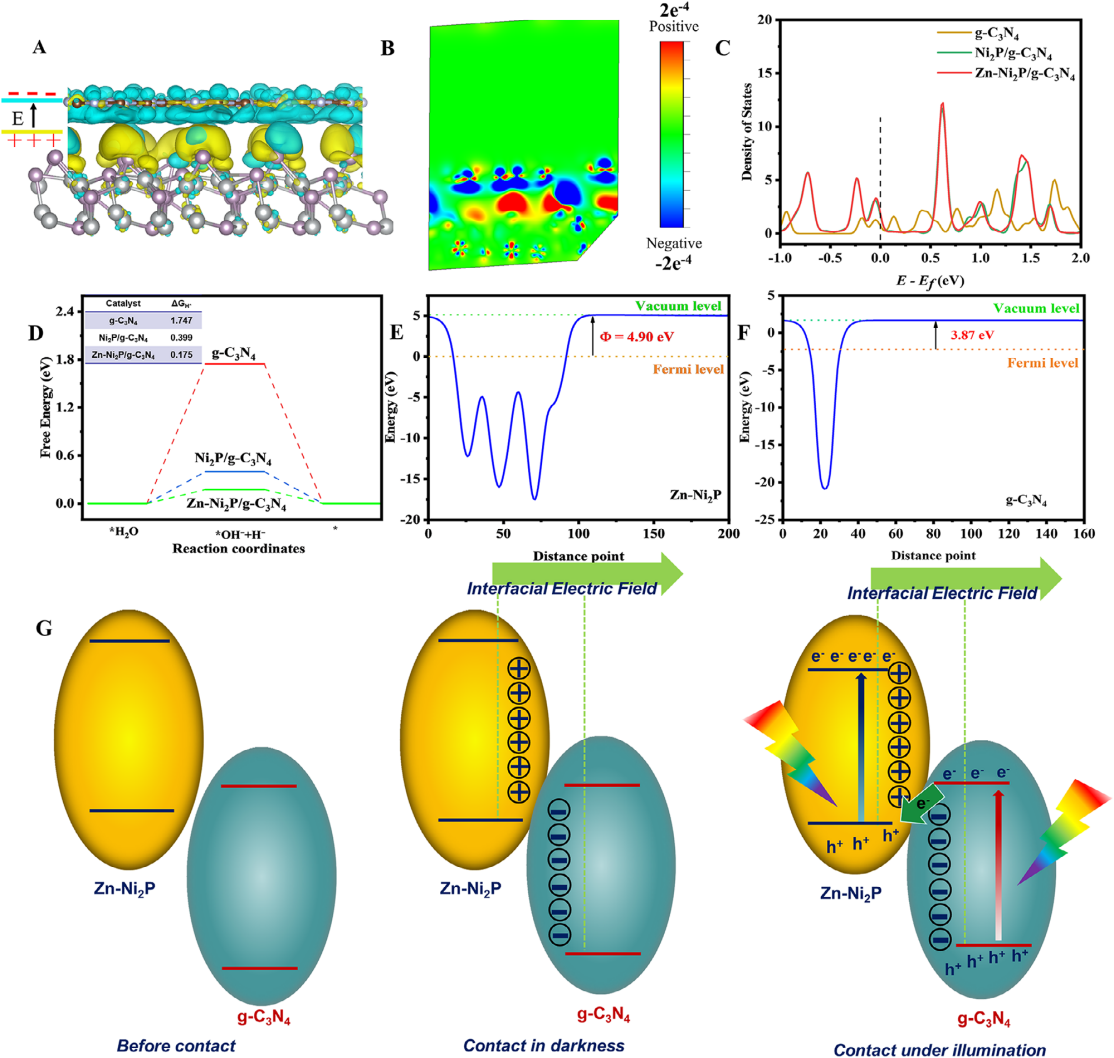
(A) Charge density difference of the 3D‐optimized structure model, (B) the corresponding longitudinal section image of Zn‐Ni_2_P and g‐C_3_N_4_, (C) p‐band density of states of g‐C_3_N_4_, Ni_2_P/g‐C_3_N_4_, and Zn‐Ni_2_P/g‐C_3_N_4_. (D) Hydrogen adsorption Gibbs free energy of g‐C_3_N_4_, Ni_2_P/g‐C_3_N_4_, and Zn‐Ni_2_P/g‐C_3_N_4_, Computational work function of (E) Zn‐Ni_2_P and (F) g‐C_3_N_4_. (G) The energy band arrangements of Zn‐Ni_2_P and g‐C_3_N_4_ before contact, after contact in darkness, and contact under illumination.

Density functional theory (DFT) further reveals the electronic structure information of Zn‐Ni_2_P/g‐C_3_N_4_ and the charge transfer trend of the two‐phase contact interface. Based on the model in Figure S5, the electronic states presented by the nanostructure of Zn‐Ni_2_P/g‐C_3_N_4_ are discovered by DFT calculation. Charge distribution is reflected by differential charge density (Figure 6A,B), and it is seen that charges distribute severely unevenly on Zn‐Ni_2_P/g‐C_3_N_4_, in which positive charges mainly gather in Zn‐Ni_2_P (yellow color) and negative in g‐C_3_N_4_ (cyan color), especially near the triangular caves (Figure S6). The above results show a strong electron enrichment capability on the Zn‐Ni_2_P side of the composites [32]. Due to the large charge density difference between Zn‐Ni_2_P and g‐C_3_N_4_, an IEF from Zn‐Ni_2_P to g‐C_3_N_4_ is formed at the interface of the Zn‐Ni_2_P/g‐C_3_N_4_ heterojunction, as shown in Figure 6A. In the meantime, the band gap can also be observed by the density of states (DOS), which is consistent with the band structures of Zn‐Ni_2_P and g‐C_3_N_4_ [59, 60]. Additionally, the longitudinal section image of differential charge density can more visually display the charge density difference between g‐C_3_N_4_ and Zn‐Ni_2_P charge density difference (Figure 6C). These findings are consistent with the XPS analysis results (Figure 3). Figure 6C shows the DOS for g‐C_3_N_4_, Zn‐Ni_2_P, Ni_2_P/g‐C_3_N_4_, and Zn‐Ni_2_P/g‐C_3_N_4_. Both Zn‐Ni_2_P and g‐C_3_N_4_ exhibit intermittent DOS, indicating semiconducting properties. g‐C_3_N_4_ has a much lower DOS near the Fermi level compared to other catalysts, resulting in a low carrier density and limiting charge transfer, which is consistent with transient photocurrent and EIS measurements [61, 62]. Besides, the DOS peak strength can reflect the H* adsorption strength of the catalyst, and the p band density of Zn‐Ni_2_P/g‐C_3_N_4_ increases with the addition of Zn‐Ni_2_P, which is conducive to H* adsorption [63]. The Gibbs free energy of hydrogen adsorption (∆*G*_H*_) reflects the photocatalyst's ability to adsorb and desorb H* during the HER process, and it is a key factor influencing HER performance. As shown in Figure 6D, Zn‐Ni_2_P/g‐C_3_N_4_ exhibits the lowest ∆*G*_H*_ value (0.175 eV) among the tested catalysts. This indicates its superior H* adsorption/desorption capability in the HER process. This is entirely consistent with the DFT calculation of DOS. The aforementioned study indicated that the incorporation of Zn‐Ni_2_P into g‐C_3_N_4_ not only promotes the upshift of the p‐band density of state in Zn‐Ni_2_P/g‐C_3_N_4_, favorable for the H* adsorption, but also considerably accelerates the photogenerated charge separation by a strong IEF on the heterointerface. To further understand the electron transfer between g‐C_3_N_4_ and Zn‐Ni_2_P and elucidate the endogenic dynamics of photogenerated charge migration, the work functions (Φ) of g‐C_3_N_4_ and Zn‐Ni_2_P were calculated theoretically using DFT. As shown in Figure 6E,F, the functions of g‐C_3_N_4_ and Zn‐Ni_2_P are 3.87 and 4.90 eV, respectively. Normally, when two semiconductors with different work functions form a heterojunction, electrons flow from one of the lower work functions to the other. Therefore, photogenerated charges in the Zn‐Ni_2_P/g‐C_3_N_4_ heterojunction will migrate from g‐C_3_N_4_ to Zn‐Ni_2_P under visible light irradiation. This charge redistribution and high interfacial chemical interaction can induce and form an IEF between Zn‐Ni_2_P/g‐C_3_N_4_. Under visible light, g‐C_3_N_4_ and Zn‐Ni_2_P are stimulated, and IEF and band bending motivate photoproduced e‐migration from Zn‐Ni_2_P to g‐C_3_N_4_. The formation process of a heterojunction is shown in Figure 6G. Consequently, the Z‐scheme heterojunction between g‐C_3_N_4_ and Zn‐Ni_2_P was successfully fabricated [64, 65, 66].

**FIGURE 7:**
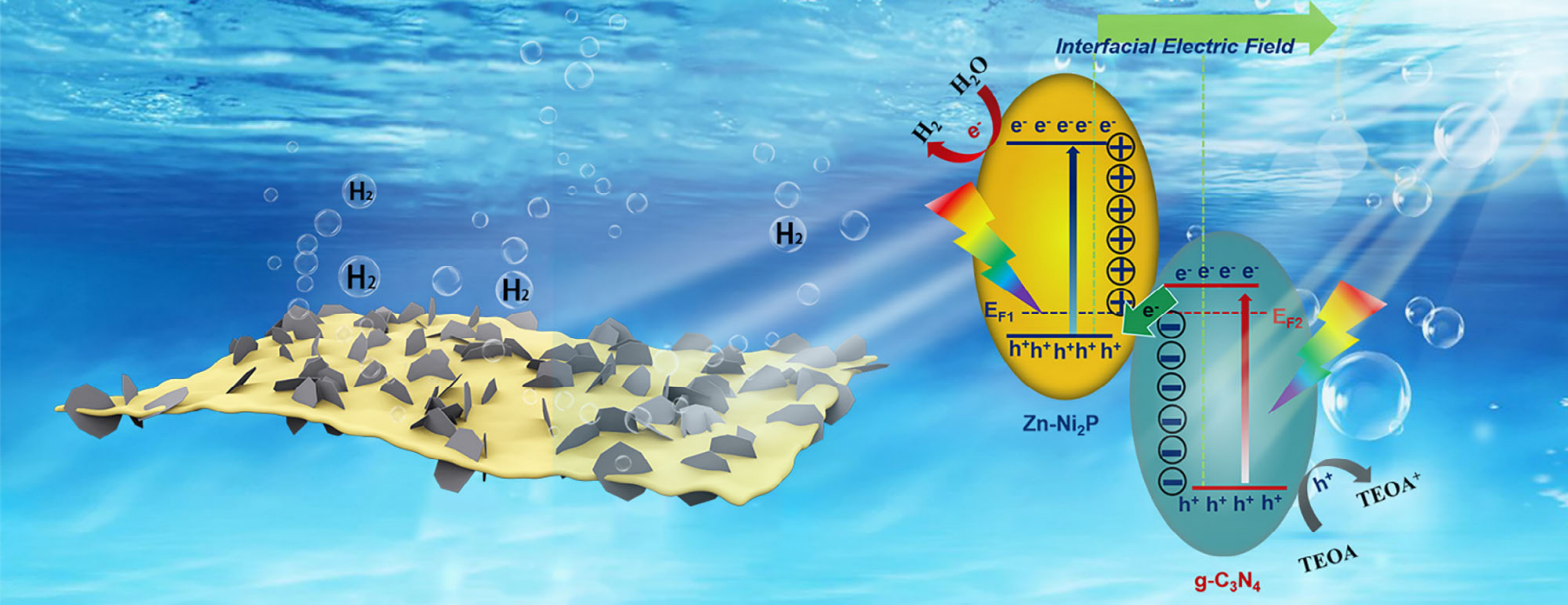
Proposed mechanism for photocatalytic H_2_ evolution in the Zn‐Ni_2_P/g‐C_3_N_4_ system under visible‐light irradiation.

Based on these experimental results and DFT calculations, we propose a photocatalytic HER mechanism for Zn‐Ni_2_P/g‐C_3_N_4_, as illustrated in Figure 7. As a typical n‐type semiconductor, the photogenerated carriers on the surface of g‐C_3_N_4_ easily recombination. To address this issue, Zn‐Ni_2_P is incorporated into g‐C_3_N_4_ to form the Z‐scheme heterojunction, where photogenerated electrons transfer from g‐C_3_N_4_ to Zn‐Ni_2_P at the interface, assisted by the work function and a strong internal electric field (IEF), as confirmed by XPS and DFT calculations. Then, Zn‐Ni_2_P captures electrons and active H* species to convert them into H_2_, with the H* species more readily adsorbing on the edge of Zn‐Ni_2_P (Figure S7) [56, 67]. From a structural perspective, the Zn‐Ni_2_P/g‐C_3_N_4_ composite has a larger heterogeneous interface. This provides more reaction sites and enhances the separation of photogenerated charges. As a result, the composite photocatalyst shows faster reaction kinetics for HER.

## 4 Conclusions

In summary, we successfully synthesized the Zn‐Ni_2_P/g‐C_3_N_4_ Z‐scheme heterojunction with excellent photocatalytic HER performance under visible light. The relationship between the composition, structure, and performance of photocatalysts is discussed through a variety of structural characterizations, performance tests, and theoretical calculations. For the Zn‐Ni_2_P/g‐C_3_N_4_, Zn‐doping effectively limits the size of the Ni_2_P nanosheets. Additionally, due to the well‐integrated Z‐scheme heterojunction of Zn‐Ni_2_P and g‐C_3_N_4_, Zn‐Ni_2_P/g‐C_3_N_4_ shows abundant active sites, enhanced light absorption, and strong electron coupling interactions. These features significantly contribute to the improved photocatalytic HER activity of Zn‐Ni_2_P/g‐C_3_N_4_. Notably, experimental results and DFT calculations demonstrate that the construction of the Z‐scheme heterostructure in Zn‐Ni_2_P/g‐C_3_N_4_ promotes the generation of IEF directing from Zn‐Ni_2_P to g‐C_3_N_4_, with the work function accelerating the photogenerated charge separation. The upshift of the p‐band density of state in Zn‐Ni_2_P/g‐C_3_N_4_ further favors H* adsorption for photocatalytic HER. Under visible light, the optimized Zn‐Ni_2_P/g‐C_3_N_4_ photocatalyst exhibits excellent hydrogen evolution performance (1077 µmol g^−1^ h^−1^), surpassing even Pt/g‐C_3_N_4_ (538 µmol·g^−1^·h^−1^), along with long‐term stability. This work provides a new strategy for enhancing the separation of photogenerated charges to improve photocatalytic properties in solar‐driven materials and devices.

## Conflicts of Interest

The authors declare no conflicts of interest.

## Supporting information




**Supporting Figure 1**: exp270072‐sup‐0001‐SuppMat.docx

## Data Availability

The data that support the findings of this study are available from the corresponding author upon reasonable request.
