# Supplementary material for: Accelerated Photogenerated Charge Separation Driven Synergistically by the Interfacial Electric Field and Work Function in Z‐Scheme Zn‐Ni2P/G‐C3N4 for Efficient Photocatalytic Hydrogen Evolution
Source: Exploration (Beijing). 2025 Aug 5;5(5):20240189. doi: 10.1002/EXP.20240189 (PMC12561414; doi:10.1002/EXP.20240189)
Supplement: Supplementary file 1 — Supporting Figure 1: exp270072‐sup‐0001‐SuppMat.docx [file EXP2-5-20240189-s001.docx]

Supporting Information

Accelerated photogenerated charge separation driven synergistically by the interfacial electric field and work function in Z-scheme Zn-Ni_2_P/g-C_3_N_4_ for efficient photocatalytic hydrogen evolution

*Qian Chen^a^, Jianfeng Huang^a, *^, Dewei Chu^b^, Liyun* *Cao^a, *^, Xiaoyi Li ^a^, Yong Zhao ^a, c^, Yijun Liu^d^, Junle Dong^c^, Liangliang Feng^a, *^*

*^a^ School of Materials Science and Engineering, International S&T Cooperation Foundation of Shaanxi Province, Xi’an Key Laboratory of Green Manufacture of Ceramic Materials, Shaanxi University of Science and Technology, Xi’an 710021, China*

*^b^ School of Materials Science and Engineering, The University of New South Wales, Sydney, NSW 2052, Australia*

*^c^ Guangxi Monalisa New Material Co., LTD; Wu Zhou, Guangxi 543300, PR China*

*^d^ Guangdong Mona Lisa Group Co., Ltd., Foshan, Guangdong 528211, PR China*

*E-mail: huangjf@sust.edu.cn (J. Huang), caoliyun@sust.edu.cn (L. Cao), fengll@sust.edu.cn (L. Feng)*

Number of pages: 14
Number of figures: 7
Number of tables: 1

**Containing 14 pages, 7 Figures, and 1 table**

**1 Experimental**

**1.1 Materials**

All chemical reagents were of analytical purity and used directly without further purification, including melamine, NiCl_2_·6H_2_O, Zn(CH_3_COO)_2_, urea, methanol, and NaH_2_PO_2_·6H_2_O.

**1.2 Experimental**

*1.2.1 Synthesis of the g-C_3_N_4_ nanosheets*

g-C_3_N_4_ is generated by melamine pyrolysis in the air. 10 g of melamine is tiled in a porcelain boat, then placed in a muffle furnace and kept for four hours at 550 °C. After natural cooling, the product was ground, then washed with deionized water and ethanol more than six times, and dried at 60 °C for 12 hours. The resulting pale yellow powder is labeled g-C_3_N_4_.

*1.2.2 Synthesis of the NiZn-LDH*

Zn(AC)_2_·2H_2_O (0.1 mmol), NiCl_2_·6H_2_O (0.45 mmol), and urea (3 mmol) in turn were dissolved in 60 mL methanol solution (V_water_: V_methanol_ = 2:3), and then stirred for 30 min at room temperature. Afterward, the solution was poured into a 100 mL Teflon-lined stainless-steel autoclave and continued at 170 °C oven for 17 hours. After natural cooling, precipitate by centrifugal separation, then wash with deionized water and ethanol more than six times, drying at 60 °C for 12 hours. The resulting light green powder is labeled NiZn-LDH.

*1.2.3 Synthesis of the NiZn/g-C_3_N_4_*

NiZn/g-C_3_N_4_ is prepared similarly to NiZn-LDH. Zn(AC)_2_·2H_2_O (0.1 mmol), NiCl_2_·6H_2_O (0.45 mmol), urea (3 mmol) and g-C_3_N_4_ (50 mg, 75 mg, and 100 mg) in turn were dissolved in 60 mL methanol solution (V_water_: V_methanol_ =2:3), and then stirred for 30 min at room temperature. Afterward, the solution was poured into a 100 mL Teflon-lined stainless-steel autoclave and continued at 170 °C oven for 17 hours. After natural cooling, the sediment was precipitated by centrifugal separation, followed by washing with deionized water and ethanol more than six times, and drying at 60 °C for 12 hours. The resulting powder is labeled NiZn/g-C_3_N_4_-1:1, NiZn/g-C_3_N_4_-1:2, NiZn/g-C_3_N_4_-1:3, and NiZn/g-C_3_N_4_-1:4.

*1.2.4 Synthesis of the Ni/g-C_3_N_4_*

Ni/g-C_3_N_4_ is prepared similarly to NiZn/g-C_3_N_4_. NiCl_2_·6H_2_O (0.45 mmol), urea (3 mmol), and g-C_3_N_4_ in turn were dissolved in 60 mL methanol solution (V_water_: V_methanol_ =2:3), and then stirred for 30 min at room temperature. Afterward, the solution was poured into a 100 mL Teflon-lined stainless-steel autoclave and continued at 170 °C oven for 17 hours. After natural cooling, the sediment was precipitated by centrifugal separation, followed by washing with deionized water and ethanol more than six times and drying at 60 °C for 12 hours. The resulting powder is labeled Ni/g-C_3_N_4_.

*1.2.5 Synthesis of the Zn-Ni_2_P*

The Zn-Ni_2_P composite photocatalyst was fabricated by a typical calcination method. Firstly, the porcelain boat is placed upstream and downstream, respectively phosphorus source and the resulting powder (NiZn-LDH), and then placed in the center of the tube furnace. Particularly, the mass ratio of phosphorus sources and powders is (3:5). The samples were heated to 300 °C or 500 °C with a heating speed of 5 °C min^-1^ in a hydrogen and argon mixed atmosphere (5% H_2_) for 2 hours. The product was collected after cooling down to room temperature under the protection gas of 5% H_2_/Ar, the product was ground, then washed with deionized water and ethanol more than six times, and dried at 60 °C for 12 h. The resulting black powder is labeled Zn-Ni_2_P.

*1.2.6 Synthesis of the Zn-Ni_2_P/g-C_3_N_4_*

Zn-Ni_2_P/g-C_3_N_4_ is prepared similarly to Zn-Ni_2_P. Firstly, the porcelain boat is placed upstream and downstream, respectively phosphorus source and the resulting powder (NiZn/g-C_3_N_4_), and then placed in the center of the tube furnace. Particularly, the mass ratio of phosphorus sources and powders is (3:5). The samples were heated to 300 °C or 500 °C with a heating speed of 5 °C min^-1^ in a hydrogen and argon mixed atmosphere (5% H_2_) for 2 hours. The product was collected after cooling down to room temperature under the protection gas of 5% H_2_/Ar. The product was ground, then washed with deionized water and ethanol more than six times, and dried at 60 °C for 12 hours. The resulting black powder is labeled Zn-Ni_2_P/g-C_3_N_4_.

*1.2.7 Synthesis of the Ni_2_P/g-C_3_N_4_*

Ni_2_P/g-C_3_N_4_ is prepared similarly to Zn-Ni_2_P/g-C_3_N_4_. Firstly, the porcelain boat is placed upstream and downstream, respectively phosphorus source and the resulting powder (Ni/g-C_3_N_4_), and then placed in the center of the tube furnace. Particularly, the mass ratio of phosphorus sources and powders is (3:5). The samples were heated to 300 °C or 500 °C with a heating speed of 5 °C min^-1^ in a hydrogen and argon mixed atmosphere (5% H_2_) for 2 h. The product was collected after cooling down to room temperature under the protection gas of 5% H_2_/Ar. The product was ground, then washed with deionized water and ethanol more than six times, and dried at 60 °C for 12 hours. The resulting black powder is labeled Ni_2_P/g-C_3_N_4_.

**1.3 Characterizations**

The phase structures of samples were recorded by using a Rigaku, D/max-2200pc X-ray diffractometer (XRD, Cu Kα1 radiation, λ = 0. 15406 nm, scan speed: 10°·min^-1^) with the 2θ range from 10° to 80°. Field emission scanning electron microscope (FESEM, Hitachi, S-4800), and high-resolution transmission electron microscopy (HRTEM, Tecnai G2 F20S-TWIN), respectively. The X-ray photoelectron spectroscopy (XPS) is conducted on a Surface Science Instruments Spectrometer with a monochromatic Al Kα source. The Ultraviolet-visible (UV-vis) absorption spectra are obtained on Cary 5000 UV-vis spectrometer (Agilent). The photoluminescence (PL) spectra and time-resolved photoluminescence decay spectra are measured on the Edinburgh FS5 spectrophotometer.

**1.4 electrocatalytic activity for H_2_ evolution**

The electrocatalytic HER activity of photocatalysts is evaluated using a three-electrode system in an alkaline medium (1.0 M KOH) and the loading on the glassy carbon electrode was about 0.714 mg/cm^2^.

**1.5 Photocatalytic activity for H_2_ evolution**

The photocatalytic reaction was carried out in a quartz flask equipped with a flat optical entry window. 50 mg of the photocatalyst was dispersed in 100 mL of an aqueous solution containing 15% triethanolamine (TEOA) in volume as a sacrificial agent. A 300-W Xe lamp with a cut-off filter (λ ≥ 420 nm) was used as the visible-light source. The amount of H_2_ evolved was determined at an interval of 1 h using an online gas chromatography system (GC-7920). The apparent quantum efficiency (AQE) was measured under the same photocatalytic reaction conditions on irradiating by using a 300-W Xe lamp with a 420 nm band pass filter. The photon flux of the incident light was determined using an optical power meter. In the AQE test, the reaction mixtures were irradiated for 60 min. AQE was calculated using the following equation:

$$AQE\left( \% \right)=\frac{number of reacted electrons}{number of incident photons}=\frac{number of evolved H molecules*2}{number of incident photons}$$

**1.6 Theoretical calculations**

All calculations were carried out based on density functional theory (DFT) as implemented in the Vienna ab initio simulation package (VASP) with the exchange-correlation functional of generalized gradient approximation (GGA) of Perdew, Burke, and Ernzerhof (PBE) method. A grid of 1 × 1 × 1 Monkhorst−Pack k-points was used for the structural relaxation.

A vacuum layer of 15 Å is adopted in the direction perpendicular to the monolayer surface to avoid the interactions between periodic slabs. The energy cutoff was set to be 450 eV. The convergence criterion for the energy and maximum force for the optimization was set to 10^-5^ eV and 0.05 eV/Å, respectively. The adsorption ability with the surface was evaluated by comparing the adsorption energy, the adsorption energy is defined as:

∆ *E*_ads_ = *E*_adsorb/surf_ − *E*_surf_ – *E*_adsorb_

Where *E*_adsorb/surf_, *E*_surf_, and *E*_adsorb_ are the calculated total energies of the substrate with adsorbate(s), the clean substrate, and the isolated adsorbate, respectively.

The result of the simple kinetic model is now plotted as a function of the free energy for hydrogen adsorption,

*∆G*_H*_ = ∆*E*_ads_ + 0.24 eV

**2 Additional Images and Data**

**Figure S1** XRD patterns of (A) Ni_2_P/g-C_3_N_4_ and (B) Ni_2_P.

**Figure S2** SEM image of (A) g-C_3_N_4_ and (B) Zn-Ni_2_P, TEM image of (C) Zn-Ni_2_P/g-C_3_N_4_, (D) SEM image, (E) TEM image of Ni_2_P/g-C_3_N_4_.

**Figure S3** The corresponding photocatalytic H_2_ evolution rates of Zn-Ni_2_P/g-C_3_N_4_ with different Zn-Ni_2_P load ratios.

**Figure S4** (A) SEM image, (B) TEM image, (C) High-resolution TEM image, (D) XRD patterns and XPS spectra of Zn-Ni_2_P/g-C_3_N_4_ after test.

**Figure S5** The calculation modes of Zn-Ni_2_P/g-C_3_N_4_.

**Figure S6** Charge density difference of the 3D-optimized structure model of Zn-Ni_2_P/g-C_3_N_4_.

**Figure S7** Adsorption mode of water dissociation intermediate (H*) on Zn-Ni_2_P/g-C_3_N_4_.

**Table S1** Summary of the Photocatalytic H_2_ Evolution on g-C_3_N_4_-Based Photocatalysts.


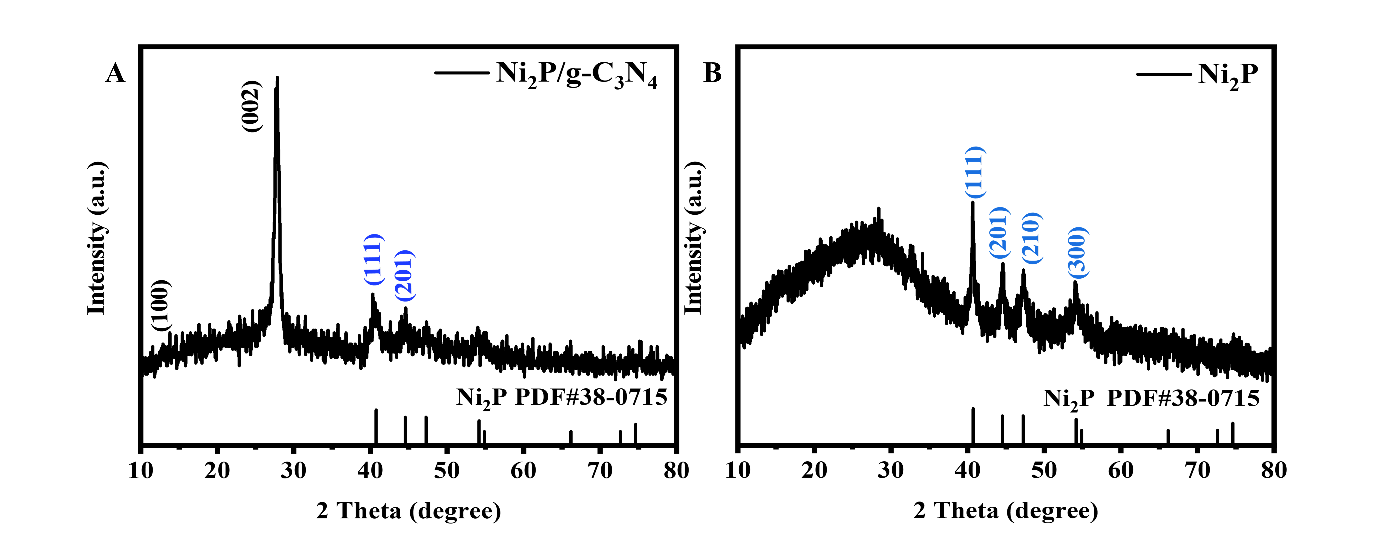


**Figure S1** XRD patterns of (A) Ni_2_P/g-C_3_N_4_ and (B) Ni_2_P.


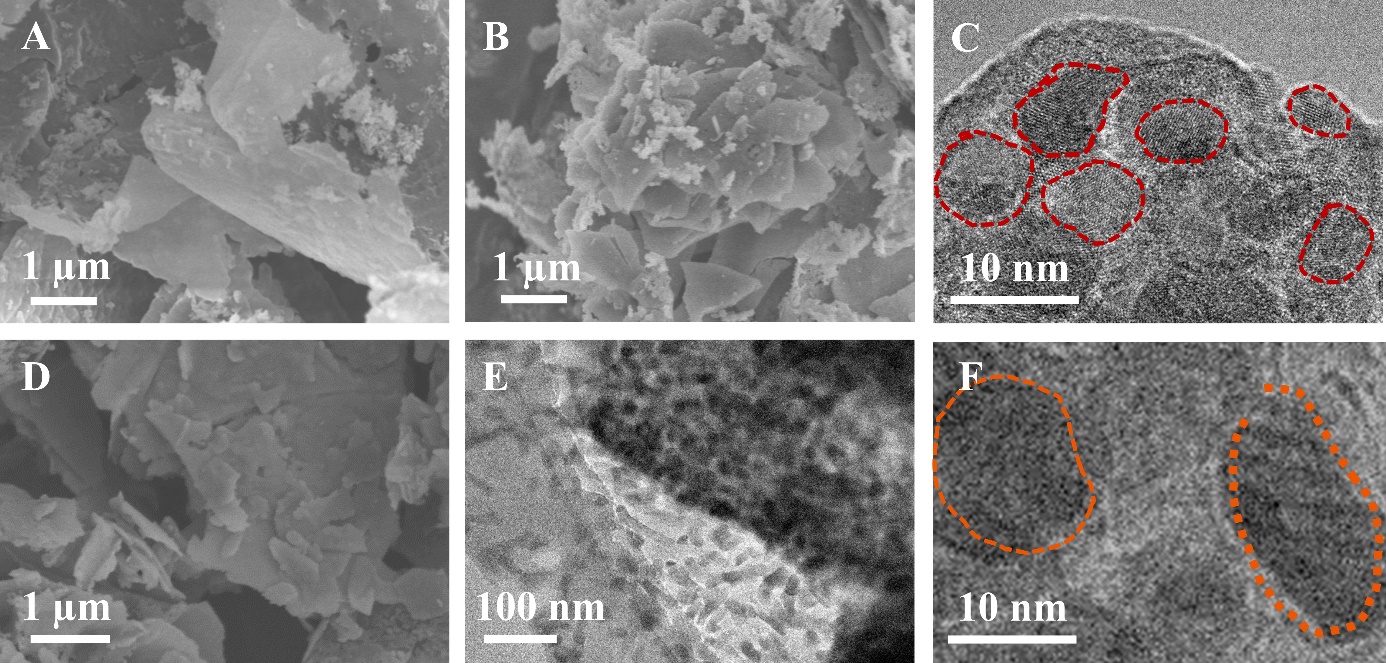


**Figure S2** SEM image of (A) g-C_3_N_4_ and (B) Zn-Ni_2_P, TEM image of (C) Zn-Ni_2_P/g-C_3_N_4_, (D) SEM image, (E) and (F) TEM image of Ni_2_P/g-C_3_N_4_.


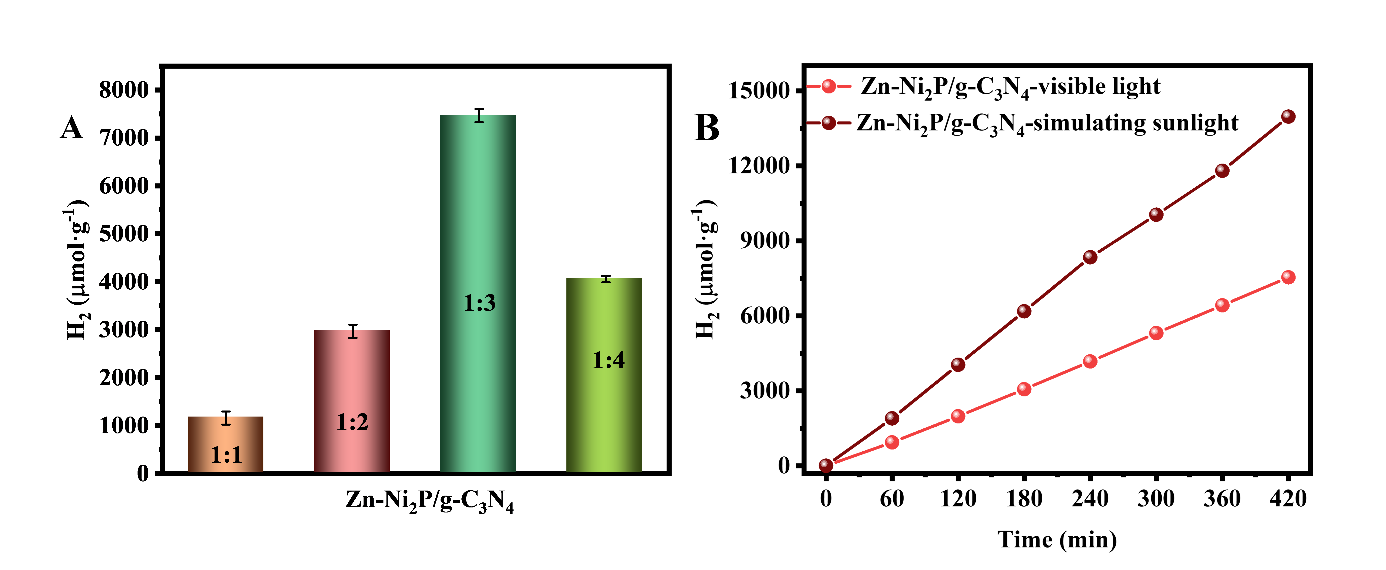


**Figure S3** (A) The corresponding photocatalytic H_2_ evolution rates of Zn-Ni_2_P/g-C_3_N_4_ with different Zn-Ni_2_P load ratios, (B) Time courses of photocatalytic H_2_ production of Zn-Ni_2_P/g-C_3_N_4_ under visible light irradiation and simulating sunlight.


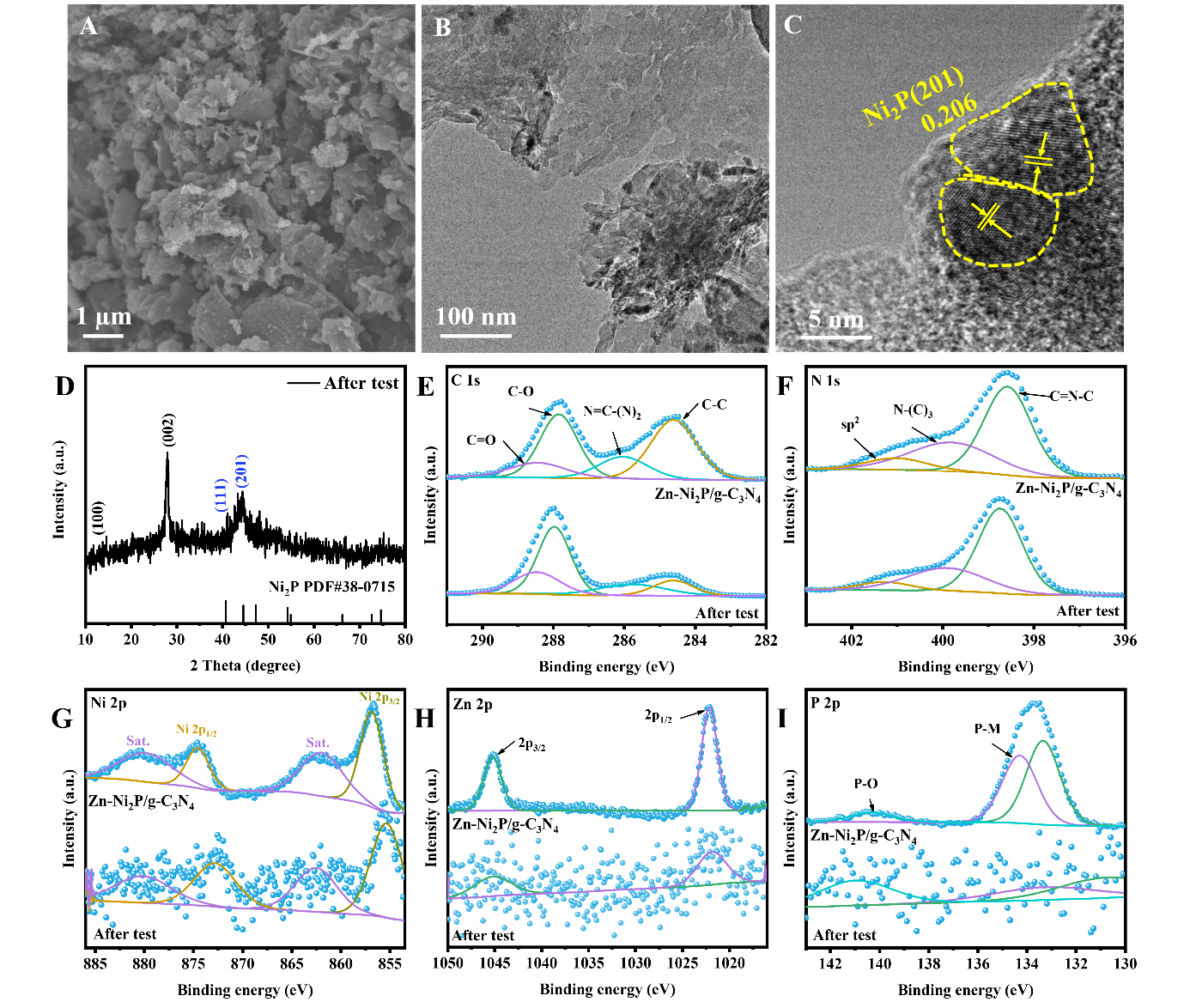


**Figure S4** (A) SEM image, (B) TEM image, (C) High-resolution TEM image, (D) XRD patterns and XPS spectra of Zn-Ni_2_P/g-C_3_N_4_ after test.


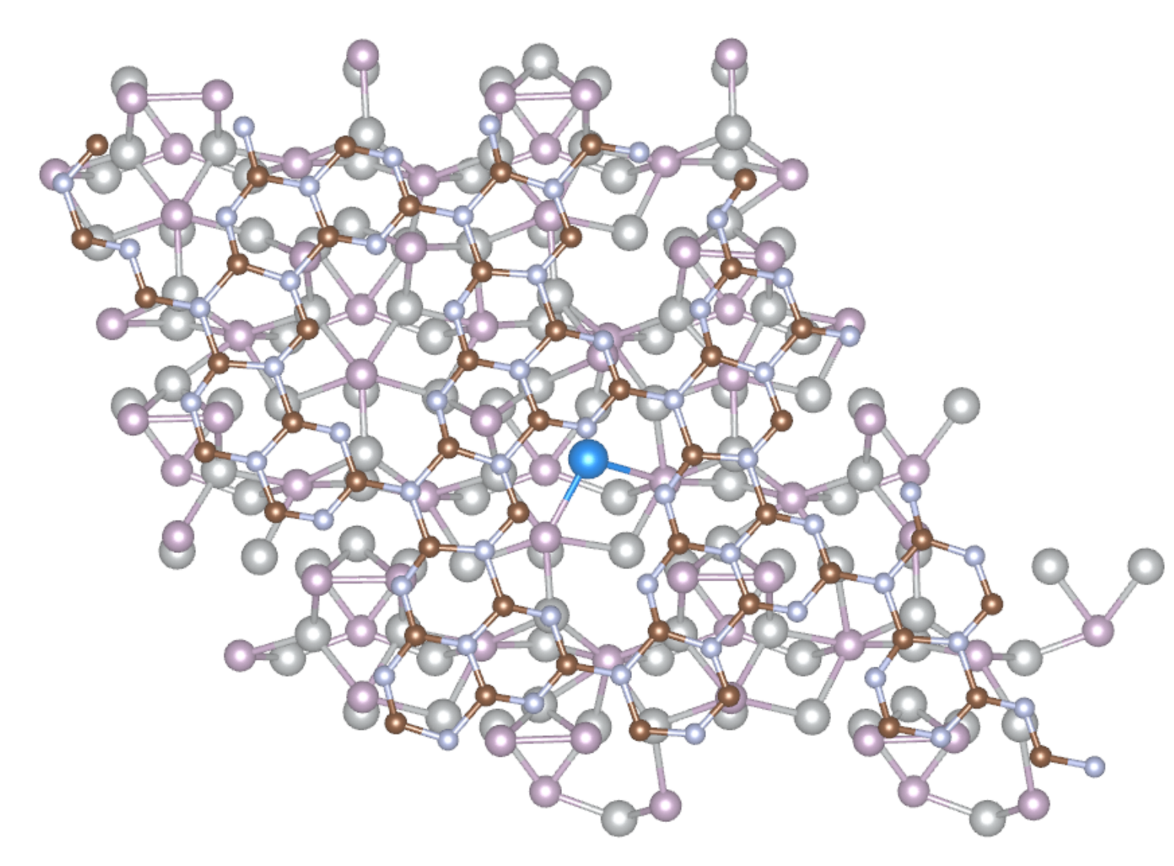


**Figure S5** The calculation modes of Zn-Ni_2_P/g-C_3_N_4_.


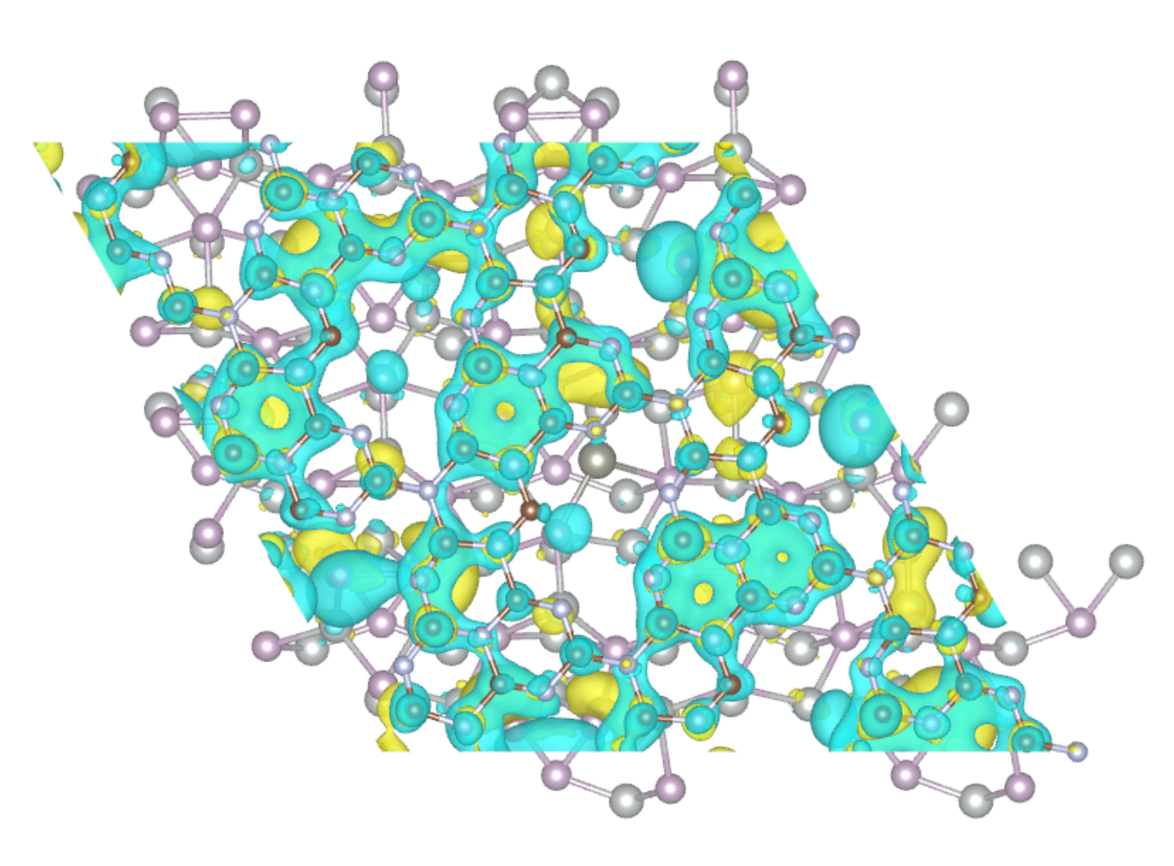


**Figure S6** Charge density difference of the 3D-optimized structure model of Zn-Ni_2_P/g-C_3_N_4_.


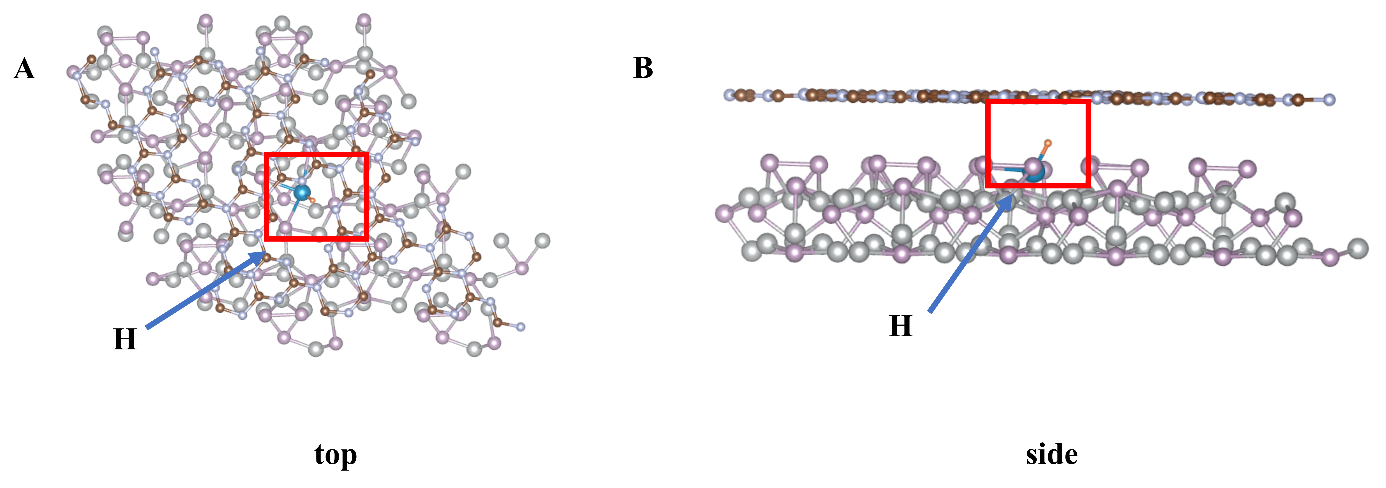


**Figure S7** The adsorption mode of water dissociation intermediate (H*) on Zn-Ni_2_P/g-C_3_N_4_.

**Table S1.** Summary of the Photocatalytic H_2_ Evolution on g-C_3_N_4_-Based Photocatalyst.

| photocatalysts | co-catalysts | power  (Xe lamp), wavelength | activity  (μmol·h^−1^·g^−1^) | references |
| --- | --- | --- | --- | --- |
| g-C_3_N_4_ | Zn-Ni_2_P | 300 W,  *λ* > 420 nm | 1077 | This work |
| g-C_3_N_4_ | Ni_2_P | 300 W,  *λ* > 420 nm | 537 | This work |
| g-C_3_N_4_ | V-Ni_2_P | 300 W,  *λ* > 420 nm | 271.5 | Dalton Trans., 2023 52 7447–7456. |
| g-C_3_N_4_ | FeP | 300 W,  *λ* > 420 nm | 177.9 | ACS Appl. Mater. Interfaces 2019 11 5651-5660. |
| g-C_3_N_4_ | MoP | 300 W,  *λ* > 420 nm | 327.5 | Appl. Surf. Sci. 2018 462 822-830. |
| g-C_3_N_4_ | Mo-Mo_2_P | 300 W,  *λ* > 420 nm | 219.7 | Appl. Catal. B-Environ. 2019 243 27-35. |
| g-C_3_N_4_ | Ni/VN | 420 nm | 168.2 | Surf. Interfaces 2023 42 103406. |
| g-C_3_N_4_ | Ni_2_P/MoS_2_ | 460 nm | 298.1 | Appl. Surf. Sci. 2020 504 144448. |
| g-C_3_N_4_ | Ni_2_P | 420 nm | 270 | Catal. Lett. 2018 148 3741–3749. |
| g-C_3_N_4_ | Co-Ni_2_P | 420 nm | 67.6 | Int. J. Hydrogen Energy 2018 43 13284-13293. |
| g-C_3_N_4_ | Ni/Ni_2_P | 420 nm | 210 | J. Colloid Interface Sci. 2018 525 107-114. |
| g-C_3_N_4_ | NiP_2_ | 420 nm | 105 | Chinese J. Chem. Eng. 2022 43 31-39. |
| g-C_3_N_4_ | MoS_2_/ Ni_2_P | 420 nm | 298.1 | Appl. Surf. Sci. 2022 504 144448. |
| g-C_3_N_4_ | Au/Ni_2_P | 420 nm | 78.65 | Appl. Surf. Sci. 2020 517 146187 |
| g-C_3_N_4_ | Ni_2_P | 420 nm | 47.06 | Appl. Surf. Sci. 2020 517 146187 |
| g-C_3_N_4_ | 3wt%Pt/MoO_2_/Ni_2_P | 420 nm | 925.5 | ACS Sustainable Chem. Eng. 2022 10 10627–10640. |
| g-C_3_N_4_ | CdS/Ni_2_P | 420 nm | 201.32 | Appl. Catal. B Environ. Energy 2019 249 246-256. |
| g-C_3_N_4_ | Ni_2_P | 420 nm | 128.7 | Int. J. Hydrogen Energy 2023 48 15460-15472. |
| black phosphorus (BP) | Ni_2_P | 420 nm | 406.08 | ACS Appl. Nano Mater. 2022 5 13078–13089. |
| g-C_3_N_4_ | NiO/Ni_2_P | 420 nm | 504 | Chem. Eng. J. 2019 378 122161. |
